# Which religious and personal characteristics predict attitudes toward gene editing? Findings from a survey of 4,939 adults in the U.S.

**DOI:** 10.1007/s12687-026-00898-4

**Published:** 2026-05-19

**Authors:** Erin D. Solomon, Eu Gene Chin, Kari Baldwin, Lauren L. Baker, James M. DuBois

**Affiliations:** 1 Division of General Medicine & Geriatrics, Washington University, 4523 Clayton Avenue, Campus Box 8005, St. Louis, MO 63110 USA; 2 Rosemead School of Psychology, Biola University, La Mirada, CA USA

**Keywords:** Gene editing, Religious affiliation, Religious beliefs, Religious practices, Genomic medicine, Trust

## Abstract

We explored whether major religious groups’ attitudes toward gene editing differ, as well as what specific religious and personal characteristics predict attitudes toward gene editing. We administered a survey to a sample of U.S. adults (*N* = 4939), including stratified samples of nine major religious and non-religious groups. We assessed participants’ religious affiliation, beliefs, and practices, and their attitudes (i.e., support and concerns) toward gene editing. Participants generally supported gene editing but also reported moderate levels of concerns. Atheist participants reported the highest support, while Mainline Protestants reported the lowest. Muslim participants reported the highest concerns, while atheist participants reported the lowest. Views on evolution and the healthcare values of their spiritual community predicted support, while views on evolution, beliefs that one’s body is a manifestation of God, and religious discrimination predicted concerns. The strongest predictors of attitudes were distrust and genetic knowledge, with higher genetic knowledge predicting lower support. While religious affiliation predicts attitudes toward gene editing, generalizations must be avoided given the range of attitudes within each group. Additionally, education alone is unlikely to increase support for gene editing. Fostering trust among religious groups is essential to ensuring that their decisions reflect their values and priorities, rather than being driven by distrust of the healthcare system. Better understanding religion’s impact on attitudes toward gene editing may facilitate appropriate genetic counseling and inform policymaking efforts.

**Supplementary Information:**

The online version contains supplementary material available at 10.1007/s12687-026-00898-4.

## Introduction

Genomic and precision medicine technologies are rapidly advancing and provide significant opportunities for developing new treatments. One of these technologies, gene editing, involves targeting specific mutations to prevent or treat a disease or condition (Doudna and Charpentier 2014). Gene editing therapies currently exist to treat sickle cell disease, muscular dystrophy, leukemia and lymphoma, and others are currently in development to treat a myriad of health conditions (Awasthi et al. 2023; Ballantine and Tisdale 2025; Hirakawa et al. 2020; Moriyama and Yokota 2024). While advancements in gene editing technology may yield significant advances in medicine, they also generate ethical and moral questions.

Prior research on the ethical and moral considerations of gene editing has focused largely on germline gene editing, which involves edits that are passed to future generations (i.e., heritable; Evans 2020; Porteus 2015). This is in contrast to somatic gene editing, in which the gene editing only affects the individual being treated, and would not be passed to future generations (i.e., non-heritable; Evans 2020; Porteus 2015). Both raise concerns about unintended health consequences and long-term safety. For example, off-target editing events can result in unintentional edits that could cause harm, such as impairing normal cell functions or adverse developmental effects (Garden and Winickoff 2018; Guo et al. 2023). Other key ethical and moral issues include the potential to use gene editing for human enhancement, for parents to create “designer babies” in which they have selected desirable traits, and concerns over potential misuse of the technology (Delhove et al. 2020; Evans 2020; Garden and Winickoff 2018). Germline gene editing raises additional ethical concerns because individuals treated would not be able to consent their offspring to intergenerational monitoring (Cwik 2020).

Prior research has also shown that gene editing in healthcare is generally supported by both the public and genetic experts, with somatic gene editing having more support than germline gene editing, and therapeutic uses for severe conditions (e.g., Alzheimer’s disease) having more support than less severe conditions (e.g., attention deficit hyperactivity disorder; Armsby et al. 2019; Critchley et al. 2019; Delhove et al. 2020; Jedwab et al. 2020; Robillard et al. 2014). Being younger, being male, and lower education levels have also been associated with higher support for gene therapy (Hendriks et al. 2018; Jedwab et al. 2020). And while genetic experts are generally supportive of gene editing, members of the public who have higher knowledge of genetics or personal experience with genetics tend to have less favorable views toward gene editing (germline gene editing in particular; Jedwab et al. 2020).

Religion has historically played a significant role in guiding ethical and moral views (Pew Research Center 2018), but its potential impact on attitudes toward gene editing have not been substantially explored. Some scholars have argued that gene editing, particularly germline gene editing, is inconsistent with the values of certain religions including Christianity and Islam, but this work is theoretical (Cherry 2017; Lala 2020). Initial social science investigations of religion and gene editing have had mixed results. Some research has found that religious beliefs are not a major driver of attitudes toward gene editing, while others have found that religious individuals have concerns about “playing God” and an aversion to tampering with nature (Allum et al. 2014; Critchley et al. 2019; Kalidasan and Das 2022; McCaughey et al. 2019; Većkalov et al. 2023).

The majority of the U.S. public is currently affiliated with a religious group, and our recent work has shown that religion is a strong predictor of overall attitudes toward genomic medicine (DuBois et al. 2025; Gallup, 2024; Garden and Winickoff 2018; Keenan 2025). Given the relatively little research on religion’s explanatory potential for attitudes toward gene editing, we sought to explore more deeply the nature of this relationship. Our prior work (DuBois et al. 2025) investigated support for genomic and precision medicine generally (which includes not only gene editing but also activities such as biobanking, prenatal genetic testing, and stem cell research), while this research focused specifically on gene editing. This is an important endeavor because there is consensus that acceptance of genomic healthcare may increase when healthcare professionals better engage patient perspectives (Evans 2014; Glasgow et al. 2018). Further, addressing patient and family member’s concerns or religious perspectives on gene editing could be an essential element of patient-centered genetic counseling, which is non-directive and sensitive to the values and perspectives of those seeking counseling.

In this study, we administered a survey battery to a large U.S. sample that included stratified sub-samples of nine major religious and non-religious groups in the U.S. We assessed participants’ religious affiliation, the religious beliefs and practices they engaged in, and their attitudes toward gene editing. We sought to understand whether there were significant differences between groups regarding attitudes toward gene editing, and what religious beliefs and practices may predict support and concerns for gene editing. Our research questions were:

1. Does the level of support for and concerns with gene editing differ between nine major religious and non-religious groups in the U.S.?
2. What specific religious beliefs and practices predict support for and concerns with gene editing?

## Materials and methods

### Study design

We administered a survey to adults in the U.S. regarding their religious and spiritual beliefs and practices, as well as their attitudes toward gene editing (DuBois 2026). The survey contained measures assessing participants’ prayer and meditation practices, how much their religion was integrated into their daily living, religious fundamentalism, acceptance of evolution, beliefs that one’s body is a manifestation of God, beliefs that God controls everything, and the healthcare values of their spiritual community (Altemeyer and Hunsberger 2004; Hoge 1972; Mahoney et al. 2005; Pew Research Center 2014; Rutledge and Sadler 2007; Wallston et al. 1999). The survey also included the Attitudes toward Genomics and Precision Medicine (AGPM) measure, and measures of genetic knowledge, healthcare system distrust, religious discrimination, and demographics (DuBois et al. 2021; Fitzgerald-Butt et al. 2016; Furr and Kelly 1999; Harris et al. 2025; Kawika Allen et al. 2020; Shea et al. 2008).

The full AGPM measure was administered, which assesses attitudes toward several genetic and genomic issues (e.g., biobanking, prenatal genetic testing, stem cell research), one of which is gene editing. For the present research, only the gene editing items were used in the analysis. As a part of administration of the measure, the participants first read a plain language description of gene editing describing what gene editing is and what it is used for. Both somatic and germline gene editing were included in this description. Support for gene editing was assessed with one item: “*I generally support gene editing*.” Concerns with gene editing were assessed using the mean of 5 items, which cover both somatic and germline gene editing concerns : (1) “*Gene editing sounds alarming*,” (2) “*I am concerned about making any changes to genes that will be passed on to future generations*,” (3) “*I am concerned that people will undergo gene editing before potential side effects are known*,” (4) “*I think gene editing is wrong because it is like playing God*,” and (5) “*I worry that gene editing will be used to change traits that are not health related like eye color*.” Response options for both support and concern items ranged from 1 (strongly disagree) to 7 (strongly agree). The data was collected via Qualtrics (an online survey platform). Further details on the survey measures and how the survey was administered can be found in a prior publication (DuBois et al. 2025). The study was approved by the Institutional Review Board (IRB) at Washington University in St. Louis (IRB #202201153).

## Sample characteristics

We recruited participants using two survey panel services, Prolific and Cloud Research. To qualify for the study, participants needed to be 18 years or older and located in the U.S. From Cloud Research, stratified samples of at least 300 participants from each of six religious groups—Black Protestant, Catholic, Evangelical Protestant, Mainline Protestant, Jewish, and Muslim (*n* = 1940)—were recruited. From Prolific, a sample of participants who were representative of the U.S. population in terms of age, gender, and race (*n* = 2999) was recruited. All participants self-reported their religious affiliation, and were grouped into one of the six religious groups, or as atheist, agnostic, or spiritual where appropriate. The total sample size was *N* = 4939 participants. Demographics of the sample can be found in a prior publication (DuBois et al. 2025).

### Statistical analysis

We conducted two sets of analyses corresponding with our two research questions. First, we conducted two Analysis of Covariances (ANCOVAs), one predicting support for gene editing and the other predicting gene editing concerns. Second, we conducted two backward chunkwise elimination model building procedures (Kleinbaum et al. 2008), one predicting support for gene editing and the other predicting gene editing concerns. Analyses were conducted using SPSS version 29.

## Analysis of covariances (ANCOVAs)

Both ANCOVAs compared the respective outcome (i.e., support or concerns) across the nine religious and non-religious groups: (1) Black Protestant, (2) Catholic, (3) Evangelical Protestant, (4) Mainline Protestant, (5) Jewish, (6) Muslim, (7) atheist, (8) agnostic, and (9) spiritual. There were *n* = 500 participants who could not be categorized into any of these nine groups (e.g., Buddhist, Mormon) who were thus excluded from the ANCOVAs. Based on a review of prior literature, six demographic variables were selected to include as covariates: (1) age, (2) education, (3) household income, (4) urban/suburban/rural status, (5) political orientation, and (6) employment. The *Supplemental Materials* contains a description of the ANCOVA assumption checking results.

Regarding the ANCOVA predicting support for gene editing, in preliminary checks, all six covariates were significant predictors (*p*s<0.05) of support for gene editing. We assessed possible collinearity among the predictors, and excluded employment because it was highly related to age ($\:{\epsilon\:}^{2}$=0.384; Cohen 2013). A small number of participants (*n* = 123) were excluded when responses could not be used in statistical analyses, such as “prefer not to answer” or “other” income or education respectively. Thus, the final sample size in this ANCOVA was *N* = 4316.

Similarly, for the ANCOVA predicting concerns, in preliminary checks, three covariates (i.e., age, political orientation, and urban/rural status) were significant predictors (*p*s<0.05) of concerns. No multicollinearity concerns were observed between these three predictors. As in the prior ANCOVA, a few participants (*n* = 7) were excluded when responses could not be used in statistical analyses, such as “prefer not to answer” or “other.” This resulted in a final sample size of *N* = 4432.

## Model building: backward chunkwise elimination procedure

We conducted two backward chunkwise elimination procedures, one focused on support for gene editing, and the other focused on gene editing concerns. We utilized backward chunkwise elimination because it maintains theoretical integrity by grouping related variables into conceptual blocks (i.e., chunks), while using statistical criteria to exclude chunks if they fail to significantly contribute to the predictive model. To this end, we identified 28 prospective predictor variables to include in the maximum models, which could be broadly divided into four categories (demographic variables, religious group, religious or spiritual predictors, or general covariates known to predict attitudes toward gene editing in the literature; see prior publication; DuBois et al. 2025). Further details about the backward chunkwise elimination procedure can be found in the *Supplemental Materials*.

Participants who reported being atheistic or agnostic (*n* = 1008) were excluded from the models because they did not complete the measures of religious beliefs and practices. Additionally, the participants who could not be categorized into any of the religious or non-religious groups (*n* = 500) were excluded. This left a subsample of *n* = 3431 individuals, which was subsequently randomly split between a model building (training) sample (*n* = 1715) and a model reliability testing (holdout) sample (*n* = 1716).

Based on the training sample (*n* = 1715), we conducted preliminary correlations and ANOVAs between each predictor and each outcome (i.e., support and concerns) to exclude predictors that were (1) not statistically significant (*p*<.05) or (2) had a small effect size (*r*<.1; $\:{\epsilon\:}^{2}$<0.01; *R*^*2*^<0.02; Cohen 1992; Cohen 2013). For the model predicting support, these preliminary analyses suggested that eight predictors should be removed from the maximum model: (1) race, (2) ethnicity, (3) integration of religious or spiritual beliefs in daily living, (4) beliefs that one’s body is a manifestation of God, (5) belief that God controls everything, (6) private prayer frequency, and (8) private prayer time. No collinearity issues among the remaining predictors were evident in preliminary checks (e.g., *r*s$\:<$.80 between continuous predictors). Next, we sequentially removed 11 grouped predictors that produced a minimum test statistic *F*_*p*_ that was smaller than *F*_*CRIT*_ (α = 0.0032; Šidák 1967), and stopped when until the minimum test statistic *F*_*p*_ in the model was larger than *F*_*CRIT*_, leaving us with five grouped predictors. The six variables contained in these five groups were then subjected to a single variable backward elimination procedure using the same inclusion criterion (α = 0.0032). All variables met the inclusion criterion. Thus, these six predictor variables were entered into a hierarchical regression.

For the model predicting concerns, preliminary analyses suggested that nine predictors should be removed from the maximum model: (1) healthcare values of my spiritual community: support for promoting community health, (2) health in the last four weeks, (3) education, (4) household income, (5) urban, suburban, or rural status, (6) gender, (7) ethnicity, (8) employment, and (9) race. Next, we sequentially removed 10 grouped predictors that produced a minimum test statistic *F*_*p*_ that was smaller than *F*_*CRIT*_ (α = 0.0037; Šidák 1967), and stopped when the minimum test statistic *F*_*p*_ was larger than *F*_*CRIT*_, leaving us with four grouped predictors. The five variables contained in these four groups were then subjected to a single variable backward elimination procedure using the same threshold. No variables were eliminated at this final step. Thus, all five predictor variables were entered into a hierarchical regression.

## Results

Table 1 depicts the bivariate correlations between predictor variables used in the ANCOVA and backward chunkwise elimination model building procedures with both support for and concerns with gene editing.

**Table 1:** Correlations of Predictors with Support for and Concerns with Gene Editing in 4,439 Survey Participants

Predictor variable	Support	Concerns
Age	− 0.06**	− 0.04**
Education	0.11**	< 0.01
Household Income	0.12**	0.02
Political Orientation	− 0.15**	0.20**
Health in the last four weeks	− 0.12**	< 0.01
Genetic knowledge	− 0.22**	− 0.06**
Distrust towards the health care system	− 0.12**	0.35**
Private prayer frequency	− 0.08**	0.21**
Private prayer time	− 0.04*	0.24**
Meditation frequency	0.04**	0.15**
Meditation time	0.06**	0.15**
Attendance frequency in religious or spiritual group activities	0.07**	0.13**
Frequency volunteer	0.14**	0.12**
Integration of religious or spiritual beliefs in daily living	0.02	0.18**
Fundamentalist religious beliefs	− 0.06**	0.17**
Acceptance of evolution	0.21**	− 0.24**
Beliefs that one’s body is a manifestation of God	− 0.01	0.18**
Belief that they would need to conceal their religious identity from others	0.09**	0.16**
Belief that others would discriminate against them for their religion	− 0.03	0.21**
Belief that God controls everything	< 0.01	0.21**
Healthcare values of my spiritual community: permissive positions on reproductive and end of life issues	0.31**	− 0.11**
Healthcare values of my spiritual community: support for promoting community health	0.26**	− 0.08**

This table only includes scale and ordinal predictors that were used in the ANCOVA and backward chunkwise elimination model building procedure. ^**^p<.001 (2-tailed), ^*^p<.05 (2-tailed)

## ANCOVA comparing support for gene editing

**Fig. 1:**
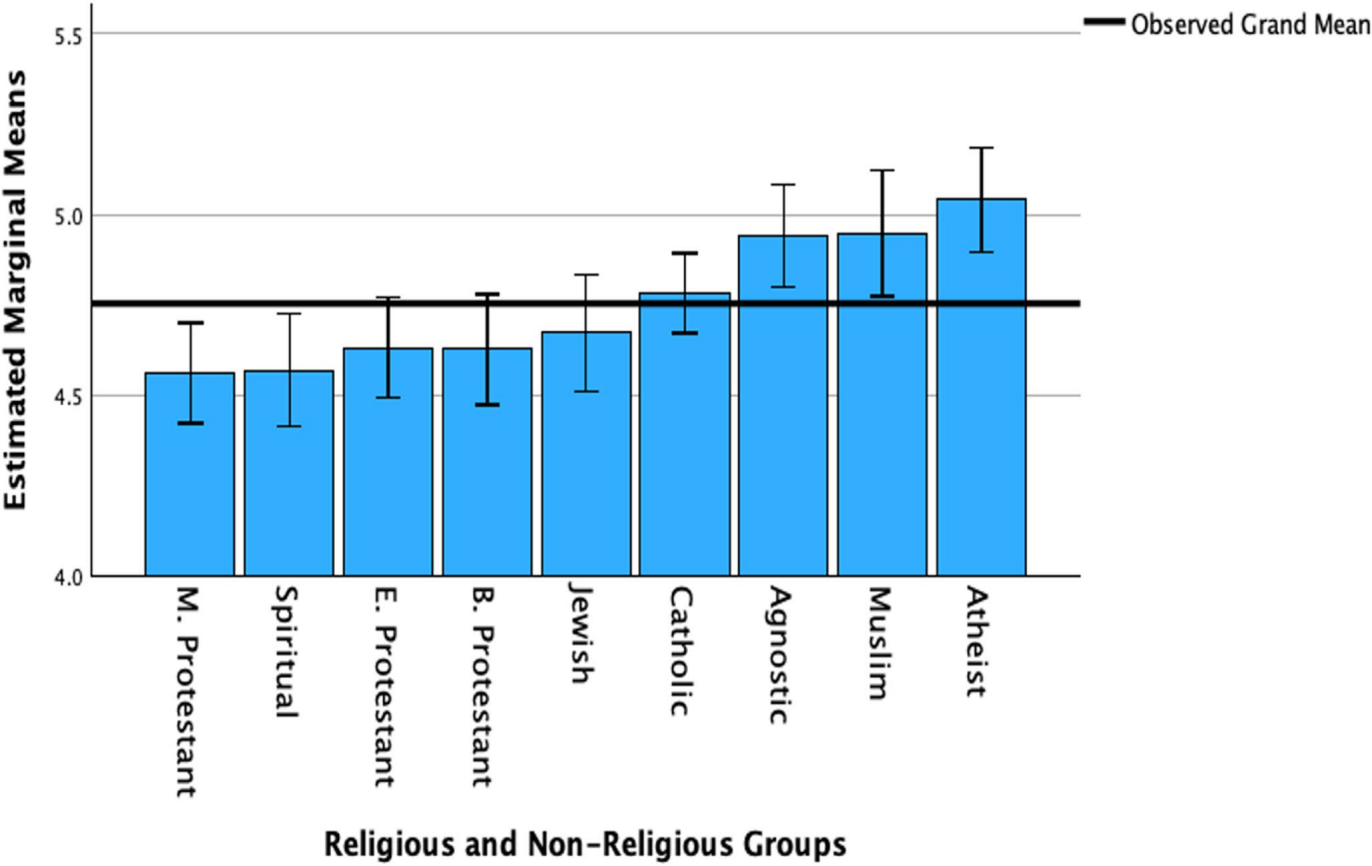
Estimated marginal means of support for gene editing among religious and non-religious groups. Note. E. Protestant = Evangelical Protestant. M. Protestant = Mainline Protestant. B. Protestant = Black Protestant. Error bars: 95% CI.

The ANCOVA results showed statistically significant, but small mean differences (partial $\:{\eta\:}^{2}$=0.010) across the religious and non-religious groups in terms of support for gene editing, *F*(8,4302) = 5.446, *p*<.001. Pairwise comparison indicated that the atheist group had significantly higher mean support for gene editing compared to the other groups ($\:\varDelta\:$*M* ranged from 0.37 to 0.48, with all *p*s<0.05)$\:,\:$except for the Catholic ($\:\varDelta\:$*M* = 0.26, *p* = .20), agnostic ($\:\varDelta\:$*M* = 0.10, *p* = 1.00), and Muslim ($\:\varDelta\:$*M* = 0.09, *p* = 1.00) groups. Figure 1 depicts the estimated marginal means and 95% Confidence Intervals across all nine religious and non-religious groups. Table 2 depicts the estimated marginal means, standard deviation and number of participants in each group.

**Table 2:** Estimated marginal means for support and concerns for gene editing among religious and non-religious groups, including standard deviations and sample size

**Support**			
**Group**	M	SD	*N*
Atheist	5.04	1.63	486
Muslim	4.95	1.62	329
Agnostic	4.94	1.61	498
Catholic	4.78	1.58	787
Jewish	4.67	1.58	364
Black Protestant	4.63	1.58	410
Evangelical Protestant	4.63	1.64	525
Spiritual	4.57	1.59	396
Mainline Protestant	4.56	1.62	521
TOTAL			4316
**Concerns**			
**Group**	***M***	***SD***	***N***
Muslim	4.97	1.31	336
Evangelical Protestant	4.76	1.34	533
Black Protestant	4.67	1.28	419
Spiritual	4.59	1.29	411
Catholic	4.56	1.29	805
Mainline Protestant	4.52	1.32	539
Jewish	4.39	1.28	381
Agnostic	4.17	1.31	510
Atheist	3.95	1.33	498
TOTAL			4432

## ANCOVA comparing gene editing concerns

**Fig. 2:**
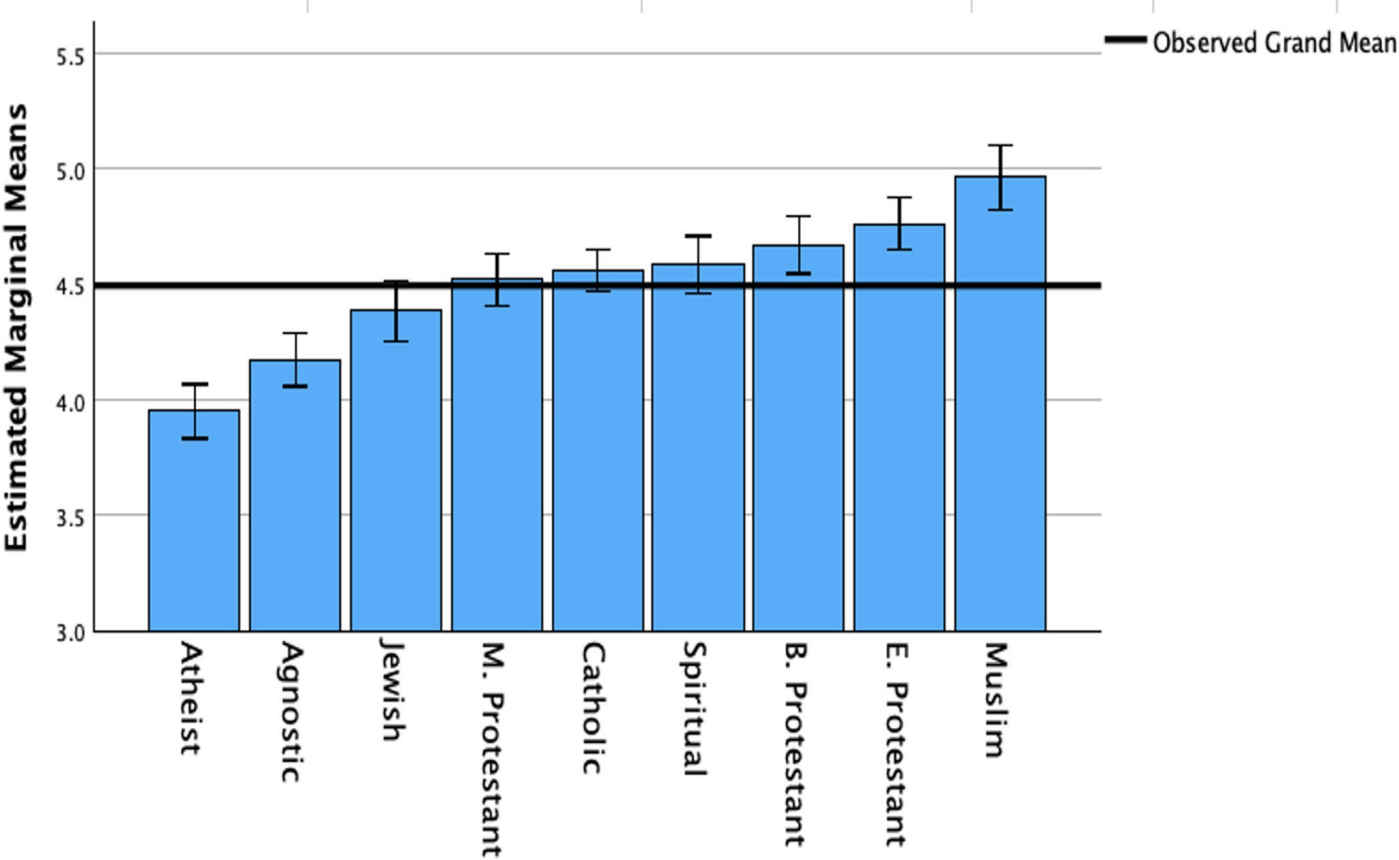
Estimated marginal means of gene editing concerns among religious and non-religious groups. Note. E. Protestant = Evangelical Protestant. M. Protestant = Mainline Protestant. B. Protestant = Black Protestant. Error bars: 95% CI

The ANCOVA was statistically significant, showing medium sized mean differences (partial $\:{\eta\:}^{2}$=0.040) across the religious and non-religious groups in terms of gene editing concerns, *F*(8,4420) = 22.36, *p*<.001. Pairwise comparisons indicated that the Muslim group had a significantly higher mean for genetic editing concerns compared to all other groups ($\:\varDelta\:$*M* ranged from 0.38 to 1.01, with all *p*s<0.05)$\:,\:$except for the Black Protestant ($\:\varDelta\:$*M* = 0.29, *p*>.05) and Evangelical Protestant groups ($\:\varDelta\:$*M* = 0.20, *p*>.05). Figure 2 depicts the estimated marginal means (accounting for covariates) across all nine religious and non-religious groups. Table 2 depicts the estimated marginal means, standard deviation, and number of participants in each group.

### Backward chunkwise elimination procedure predicting support for gene editing

We entered the six predictor variables into a hierarchical regression using the model building (training) sample (*n* = 1715), then assessed the reliability of that model using the reliability testing (holdout) sample (*n* = 1716). The cross-validation percentage relative shrinkage was − 12.15%, suggesting the model achieved an improvement of fit in the holdout sample compared to the training sample – supporting the conclusion that this was a reliable model.

Only three religious/spiritual variables remained as statistically significant predictors (*p*s<0.001) after accounting for statistically significant demographic and general covariate variables, with a small effect size (Δ*R*^*2*^=0.09; Table 3; Cohen 2013). Higher acceptance of evolution (*B* = 0.30, *p*<.001), more perceived favorable attitudes among their spiritual community pertaining to reproductive or life issues (*B* = 0.27, *p*<.001), and more perceived favorable attitudes among their spiritual community pertaining to community health (*B* = 0.29, *p*<.001) predicted higher support for gene editing. The strongest predictors of support were actually two covariates: higher genetic knowledge predicted lower support, and higher distrust toward the healthcare system also predicted lower support.

**Table 3:** Final models predicting support and concerns for gene editing

**Support**						
**Model/Predictor**	*R* ^2^	ΔR^2^	ΔF (df)	B	β	*p*
**Demographic**	0.02		30.31 (1,1680)			
Household income				0.09	0.08	< 0.001
**General Covariates**	0.09	0.07	65.87 (2,1678)			< 0.001
Genetic knowledge				-0.32	-0.22	< 0.001
Distrust towards the health care system				-0.23	-0.12	< 0.001
**Religious variables**	0.18	0.09	63.94 (3,1675)			< 0.001
Acceptance of evolution				0.30	0.17	< 0.001
Healthcare values of my spiritual community: permissive positions on reproductive and end of life issues				0.27	0.13	< 0.001
Healthcare values of my spiritual community: support for promoting community health				0.29	0.12	< 0.001
**Concerns**						
**Model/Predictor**	***R*** ^***2***^	**Δ** ***R*** ^***2***^	**Δ** ***F (*** **df)**	***B***	***β***	***p***
**General Covariates**	0.13		249.56 (1,1713)			< 0.001
Distrust towards the health care system				0.44	0.29	< 0.001
**Religious variables**	0.19	0.06	31.28 (4,1709)			< 0.001
Acceptance of evolution				-0.18	-0.13	< 0.001
Beliefs that one’s body is a manifestation of God				0.12	0.09	< 0.001
Religious discrimination: belief that they would need to conceal their religious identity from others				0.11	0.08	< 0.001
Religious discrimination: belief that others would discriminate against them for their religion				0.11	0.09	< 0.001

### Backward chunkwise elimination procedure predicting gene editing concerns

Similar to the model predicting support, for the model predicting concerns we entered the five predictor variables into a hierarchical regression using the model building (training) sample (*n* = 1715), then assessed the reliability of that model using the reliability testing (holdout) sample (*n* = 1716). The amount of variance explained in the outcome variable (*R*^*2*^) increased slightly (cross-validation percentage relative shrinkage =-0.69%), suggesting the model achieved an improvement of fit in the holdout sample compared to the training sample – supporting the conclusion that this was a reliable model.

Only four religious/spiritual variables remained as statistically significant predictors (*p*s<0.001) after accounting for one statistically significant general covariate, with a small effect size (Δ*R*^*2*^=0.06; Table 3; Cohen 2013). Specifically, stronger beliefs that one’s body is a manifestation of God (*B* = 0.12, *p*<.001), stronger belief that they would need to conceal their religious identity from others (*B* = 0.11, *p*<.001), and stronger belief that others would discriminate against them for their religion (*B* = 0.11, *p*<.001) predicted more gene editing concerns. On the other hand, higher acceptance of evolution (*B*=-0.18, *p*<.001) predicted less gene editing concerns. Additionally, similar to the prior regression model, a covariate was the strongest predictor of concerns: higher distrust toward the healthcare system predicted higher concerns.

## Discussion

This study utilized a large sample representative of the U.S. in terms of age, race, and gender with sizable sub-samples of nine religious and non-religious groups. This sampling procedure allowed for comparisons between groups on their attitudes toward gene editing which is notable because much prior research has tended to focus on religion more generally rather than examining many religious groups at once (Critchley et al. 2019; Kalidasan and Das 2022; Lala 2020; Većkalov et al. 2023). Furthermore, the study assessed a broad range of religious and spiritual beliefs and practices to see which predicted attitudes toward gene editing, rather than focusing on certain religious tenets or beliefs (Cherry 2017).

We found that participants across religious groups generally supported gene editing, which is consistent with prior work indicating general support for gene editing and genomic medicine more broadly (Armsby et al. 2019; Delhove et al. 2020; DuBois et al. 2025; Robillard et al. 2014). Participants also reported moderately high levels of concerns (which included both somatic and germline concerns), also consistent with past research (Critchley et al. 2019; Cwik 2020; Garden and Winickoff 2018; Jedwab et al. 2020). There were significant differences between religious groups on both support and concerns. For support for gene editing, atheist participants reported the highest levels of support, while Mainline Protestants reported the lowest levels, and these differences remained even when controlling for personal characteristics like age, education, income, urban/rural, and political orientation. This is consistent with past work suggesting that religious individuals show lower support for gene editing on average (Critchley et al. 2019; Većkalov et al. 2023). Regarding concerns, Muslim participants reported the highest level of concerns, and atheist participants reported the lowest levels. This aligns with prior work suggesting that gene editing may be inconsistent with the teachings of Islam due to its potential conflicts with principles of Islamic law (e.g., gene editing may cause harm, is not a customary method of treatment; Lala 2020).

The mean differences in support between the religious groups, while significant, were relatively small. In contrast, the mean differences in concerns between the religious groups were larger. There was also considerable variability within each religious group on both support and concerns. The standard deviation for each group was relatively high, and all within-group standard deviations were larger than the difference between the highest and lowest mean scores across groups. This indicates that religious affiliation alone may not be a reliable predictor of an individual’s attitudes toward gene editing, given the diverse range of attitudes present within each religious group (McCaughey et al. 2019).

The significant religious predictors of support and concerns for gene editing were different. Only acceptance of evolution was a significant predictor of both, with higher levels predicting higher support for gene editing and lower concerns. Views on evolution embodies individual’s beliefs about the relationship between science and religion/creationism, and their acceptance of science (Rutledge and Sadler 2007). It makes sense that higher scores on this measure would predict higher support and lower concerns.

The only other religious variable that predicted support was the healthcare values of their spiritual community. This included both more permissive views on reproductive and end of life issues as well as support for community health, which were both associated with higher support for gene editing. This finding suggests that religious individuals place high importance on their spiritual community’s views regarding healthcare. It could be reflective of the influence that spiritual community leaders have in shaping views of gene editing, or their religion’s interpretation of sacred texts.

Higher beliefs that one’s body is a manifestation of God predicted higher gene editing concerns. This finding echoes that of past literature suggesting that religious individuals have concerns about “playing God” or tampering with nature (Kalidasan and Das 2022; Većkalov et al. 2023). In other words, those with higher beliefs that one’s body is a manifestation of God may view gene editing as interfering with nature or God’s design of the body. They may be apprehensive about the religious or spiritual implications of manipulating genes.

Finally, the more participants endorsed experiencing religious discrimination, both beliefs that they need to conceal their religious identity and beliefs that others discriminate against them for their religion, the higher their concerns with gene editing. People who feel discriminated against because of their religion may have a general mistrust of science or scientific institutions who develop new technologies like gene editing and may thus be wary of the new technologies. Or perhaps they may feel that religious individuals’ voices are underrepresented in decision-making processes surrounding new technologies such as gene editing, and that their perspectives and interests are not adequately considered.

While these religious beliefs and practices were significantly associated with attitudes toward gene editing, the strongest predictors were genetic knowledge (the largest predictor of support) and distrust toward the healthcare system (the largest predictor of concerns). In one sense, this is unsurprising, given that these variables were included in the models because of their prior associations with attitudes toward genomic medicine. Higher distrust of the healthcare system was associated with higher concerns with gene editing. However, contrary to what might be assumed, higher genetic knowledge was associated with *lower* support for gene editing (Jedwab et al. 2020). Furthermore, the finding that these covariates predict attitudes toward gene editing more strongly than the religious variables differs from prior research showing that religious factors predicted support for genomic medicine more strongly than such covariates (DuBois et al. 2025). We also did not find any demographic differences in attitudes toward gene editing, which differs from prior work in which being younger, male, and having a lower education level were associated with higher support for gene therapy (Hendriks et al. 2018; Jedwab et al. 2020).

The relationship between higher genetic knowledge and lower support for gene editing challenges the so-called “deficit model” embraced by many experts, that is, the view that concerns with new medical technologies are largely due to ignorance (Ko 2016; Nisbet 2018). Those with higher knowledge of genetics had lower support for gene editing, indicating that simply educating the public on gene editing is unlikely to increase support. This is consistent with some past research (Jedwab et al. 2020), and may suggest that those who know more about genetics may also be more aware of the risks of gene editing than the average person. Similar patterns have been observed in other domains as well, for instance those who are more knowledgeable about internet privacy also express more concerns about privacy (Prince et al. 2023).

Additionally, distrust may be a factor that binds together variables such as rejection of evolution and religious discrimination. Prior work has suggested that rejection of evolution is often a surrogate for distrust in science, rather than a lack of scientific knowledge or a rejection of scientific beliefs; this may reflect a broader skepticism toward institutions perceived as dismissive of religion or religious views (Evans 2018). Building trust in science and the healthcare system more broadly may be the key to increasing support for gene editing, and fostering trust will require more than just education. Future research should investigate strategies for building trust.

While the public may generally be supportive of gene editing, they still have considerable concerns that may prevent them from supporting it fully. Future research should investigate when someone has the opportunity and interest in utilizing a gene editing treatment, could their concerns with gene editing be so great as to prevent them from receiving that treatment? Some studies have found that while nearly all participants report wanting to know the results of genetic tests conducted as a part of a research study, most opt not to learn their results when presented with the opportunity to do so (Henrikson et al. 2021). Will receiving gene editing therapies follow this pattern when they become more widely available? On the one hand, gene editing offers much greater promise than genetic test results alone; on the other hand, the concerns are likely much stronger given the unknown and potentially severe unintended effects (Garden and Winickoff 2018; Guo et al. 2023).

The study had some limitations. Although the large sample facilitated comparisons between several major religious groups present in the U.S., it did not allow for comparisons among smaller religious groups in the U.S. such as Mormons, Hindus, or Buddhists. Furthermore, our study approach allowed us to compare groups such as atheists and agnostics to several religious groups on their attitudes toward gene editing in the ANCOVA analyses. However, our regression analyses focused on religious predictors of attitudes toward gene editing which did not allow us to include atheist or agnostic participants, as they did not complete the religious items in the survey because it would not have been possible for them to complete such items. Another limitation was that the sample was slightly more educated and more liberal than the rest of the U.S.; though these factors were controlled for in all analyses, descriptive statistics may not be fully generalizable to the larger U.S. population and religious groups.

Because this was part of a larger study, our measures of support and concerns for gene editing were relatively brief. Support consisted of one item, and concerns consisted of one 5-item factor from the AGPM measure. While brief, our prior work validating the AGPM suggests that these measures were reliable and valid, and the concerns measure included the major concerns with gene editing (e.g., germline gene editing, “playing God,” and “designer babies”), but future research may want to use more robust assessments. Further, we tested the association of religious and other variables with attitudes; while attitudes are commonly predictors of behaviors, we cannot be sure how these variables would predict actual behavior. Lastly, the cross-sectional, non-experimental nature of research study precludes making any causal claims between our predictors and outcomes of interest.

## Conclusions

In conclusion, attitudes toward gene editing differed by religious/non-religious group, but with the caveat that there were more within group differences than between group differences when controlling for personal characteristics like age, education, and political orientation. Different religious beliefs and practices predicted attitudes toward gene editing, suggesting that support and concerns are separate constructs rather than opposite sides of the same spectrum.

Finally, we believe that several of our findings indicate that education alone will not likely lead to increases in support for gene editing. First, religious beliefs and values are often deeply held; they are not easily changed and a public health campaign aimed at changing them would be morally suspect. Second, being more knowledgeable about genetics was associated with higher levels of concern, not lower levels of concern. Finally, one of the strongest predictors of support and concerns was distrust of the healthcare system. All this suggests that building support for gene editing within religious communities may require as a first step fostering trust among religious groups. Fostering trust among religious groups is essential to ensuring that religious individuals’ decisions about genetic and genomic healthcare reflect their deeply held values and priorities, rather than distrust of the healthcare system. In the meantime, given that the majority of the U.S. public identifies with a religion, understanding religious attitudes toward gene editing is essential for informing genetic counseling which aims to respectfully engage the values and beliefs of individual patients and family members (Gallup, 2024; Garden and Winickoff 2018; Keenan 2025).

## Supplementary Information

Below is the link to the electronic supplementary material.

Supplementary Material 1

## Data Availability

The data that support the findings of this study are openly available in Open ICPSR at [https://www.openicpsr.org/openicpsr/project/244964/version/V1/view].
