# Supplementary material for: Which religious and personal characteristics predict attitudes toward gene editing? Findings from a survey of 4,939 adults in the U.S
Source: J Community Genet. 2026 May 19;17(3):64. doi: 10.1007/s12687-026-00898-4 (PMC13184050; doi:10.1007/s12687-026-00898-4)
Supplement: Supplementary file 1 — Supplementary Material 1 [file 12687_2026_898_MOESM1_ESM.pdf]

Supplemental Material

Which religious and personal characteristics predict attitudes toward gene editing? Findings from a survey of 4,939 adults in the U.S.

Journal of Community Genetics

Erin D. Solomon, Eu Gene Chin, Kari Baldwin, Lauren L. Baker, & James M. DuBois

Contact: duboisjm@wustl.edu

Table of Contents

Section	Page Number
Steps for Evaluating Reliability of Model from Backward Chunkwise Elimination Procedure	2
Backward Chunkwise Elimination Model Building Procedure	3
Assumption Checking for Analysis of Covariance (ANCOVA) Models	4
Assumption Checking for Regression Models Predicting Support for Gene Editing and Gene Editing Concerns	5
Supplemental Table 1. Maximum Model for Predicting Support for Gene Editing	6
Supplemental Table 2. Maximum Model for Predicting Gene Editing Concerns	7

Steps for Evaluating Reliability of Model from Backward Chunkwise Elimination Procedure[1]

1. After conducting the backward chunkwise elimination procedure with the training dataset, we record the R^2 vector of parameter estimates from the finalized model.
2. Next, we compute $\hat{Y}_{2|1} = X_2\hat{\beta}_1$, where X_2 is the holdout dataset. Essentially, we used the estimated prediction equation from the training group to compute predicted values of the outcome in the holdout sample.
3. Next, we compute the squared cross-validation correlation: $R_*^2(2) = r^2(Y_2, \hat{Y}_{2|1})$
4. Next, we compute the relative shrinkage: $R^2(1) - R_*^2(2)$
5. Next, we calculate the percentage relative shrinkage: $100 \times \left(\frac{R^2(1) - R^2(2)}{R^2(1)}\right)$
6. Lastly, percentage relative shrinkage values less than 0.10 suggest a model that is reliable across the training and holdout sample

Backward Chunkwise Elimination Model Building Procedure

A backward chunkwise elimination model building procedure [1] was conducted for each outcome (i.e., support for gene editing and gene editing concerns), for a total of two model building procedures, in the training group sample. The elimination procedure was first applied to "chunks" of variables (i.e., variables grouped together for conceptual similarity), then applied to individual variables that remain in "chunks" that survived the initial round of elimination. For each round of elimination, the model building procedure consisted of five steps.

1. Specify the full model as the base, with $p = k$ predictors, where k equals to the full number of chunks/variables in the model
2. Fit all p-1 variable models, defined by deleting one chunk/variable from the base model
3. For each model, compute the added-last test for the candidate chunk/variable
4. Find the minimum test statistics, $F_{p.}$
    - If statistically significant (i.e., $F_p > F_{CRIT}$, where α is based on a Sidak-Bonferroni corrected test)[2], stop and choose model p
    - If not statistically significant (i.e., $F_p < F_{CRIT}$), delete the chunk/predictor with the minimum test statistic and reduce p by 1.
5. Go back to Step 2 and repeat the process

Once a final model was determined for each outcome, we then evaluated the generalizability of the model by evaluating the previously mentioned percentage relative shrinkage value. A percentage relative shrinkage value that is less than 0.10 suggests a model is reliable across the training and holdout samples.[1]

Assumption Checking for Analysis of Covariance (ANCOVA) Models

For the ANCOVA model predicting support for gene editing, examination of residual and normal probability plots did not suggest gross violations pertaining to linearity, normality, and homoscedasticity assumptions. Examination of interaction terms between each covariate and religious and non-religious groups suggested small effect sizes (partial η^2s $\leq .012$),[3] suggesting no gross violation pertaining to homogeneity of regression slopes for the ANCOVA.

For the ANCOVA model predicting gene editing concerns, examination of residual and normal probability plots did not suggest gross violations pertaining to linearity, normality, and homoscedasticity assumptions. Examination of interaction terms between each covariate and religious and non-religious groups suggested very small effect sizes (partial η^2s $\leq .005$),[3] suggesting no gross violation pertaining to homogeneity of regression slopes.

Assumption Checking for Regression Models Predicting Support for Gene Editing and Gene Editing Concerns

For the backward chunkwise elimination procedure predicting support for gene editing, we only examined regression assumptions for the maximum model in the training sample and final model in the holdout sample.[1] Examination of residual and normal probability plots for the maximum and final models did not suggest gross violations pertaining to linearity, normality, and homoscedasticity assumptions. Tolerance, variance inflation factor, and condition indices values suggested no significant multicollinearity concerns among the predictors in the maximum and final models.

Similarly, for the backward chunkwise elimination procedure predicting gene editing concerns, we only examined regression assumptions for the maximum model in the training sample and final model in the holdout sample.[1] Examination of residual and normal probability plots for the maximum and final models did not suggest gross violations pertaining to linearity, normality, and homoscedasticity assumptions. Tolerance, variance inflation factor, and condition indices values suggested no significant multicollinearity concerns among the predictors in the maximum and final models.

Supplemental Table 1. Maximum Model for Predicting Support for Gene Editing

No.	Model/Predictor	*F*	*df*	*p*	Adjusted $R^2/\Delta R^2$
	Maximum Model				.18
1	Genetic knowledge	41.11	1	<.001	.02
2	Distrust towards the health care system	18.93	1	<.001	.01
3	Fundamentalist religious beliefs	0.43	1	.51	.00
4	Acceptance of evolution	28.73	1	<.001	.01
5	Religious discrimination: belief that they would need to conceal their religious identity from others; Religious discrimination: belief that others would discriminate against them for their religion	1.75	2	.17	.00
6	Healthcare values of my spiritual community: permissive positions on reproductive and end of life issues; healthcare values of my spiritual community: support for promoting community health	35.08	2	<.001	.03
7	Meditation frequency; meditation time	0.14	2	.87	.00
8	Attendance frequency in religious or spiritual group activities; Frequency Volunteer	0.29	2	.75	.00
9	Health in the last four weeks	1.26	1	.26	.00
10	Education level	0.01	1	.92	.00
11	Household income	3.91	1	.05	.00
12	Political orientation	1.11	1	.29	.00
13	Male, female[a], Other	3.10	2	.05	.00
14	Urban, suburban[a], or rural status	0.79	2	.46	.00
15	Employed full time[a], employed part time, caregiver, self-employed, retired, unemployed, other	0.51	6	.80	.00
16	Black Protestant, Catholic[a], Evangelical Protestant, Mainline Protestant, Jewish, Muslim, Spiritual	1.78	6	.10	.01

The full set of 20 predictors are grouped into 16 groups to accommodate dummy variables and reduce the overall number of predictors in the model.

[a] Reference categories

Supplemental Table 2. Maximum Model for Predicting Gene Editing Concerns

No.	Model/Predictor	*F*	*df*	*p*	Adjusted $R^2/\Delta R^2$
	Maximum Model				.21
1	Distrust towards the health care system	129.12	1	<.001	.06
2	Integration of religious or spiritual beliefs in daily living	0.29	1	.59	.00
3	Fundamentalist religious beliefs	3.85	1	.050	.00
4	Acceptance of evolution	12.49	1	<.001	.01
5	Beliefs that one's body is a manifestation of God	3.05	1	.08	.00
6	Religious discrimination: belief that they would need to conceal their religious identity from others; Religious discrimination: belief that others would discriminate against them for their religion	21.24	2	<.001	.02
7	Belief that God controls everything	4.18	1	.041	.00
8	Healthcare values of my spiritual community: permissive positions on reproductive and end of life issues; healthcare values of my spiritual community: support for promoting community health	7.38	2	<.001	.01
9	Private prayer frequency; private prayer time	2.92	2	.05	.00
10	Meditation frequency; meditation time	0.28	2	.75	.00
11	Attendance frequency in religious or spiritual group activities; frequency volunteer	0.17	2	.84	.00
12	Age	1.19	1	.27	.00
13	Political orientation	3.31	1	.07	.00
14	Black Protestant, Catholic[a], Evangelical Protestant, Mainline Protestant, Jewish, Muslim, Spiritual	2.55	6	.02	.01

The full set of 19 predictors are grouped into 14 groups to accommodate dummy variables and reduce the overall number of predictors in the model.
[a]Reference category

References

1. Kleinbaum DG, Kupper LL, Nizam A, Muller KE. *Applied Regression Analysis and Other Multivariable Methods*. 4th ed. Duxbury Press; 2008.

2. Šidák Z. Rectangular Confidence Regions for the Means of Multivariate Normal Distributions. *Journal of the American Statistical Association*. 1967;62(318):626-633. doi:10.1080/01621459.1967.10482935

3. Cohen J. A power primer. *Psychological Bulletin*. 1992;112(1):155-159. doi:10.1037//0033-2909.112.1.155
